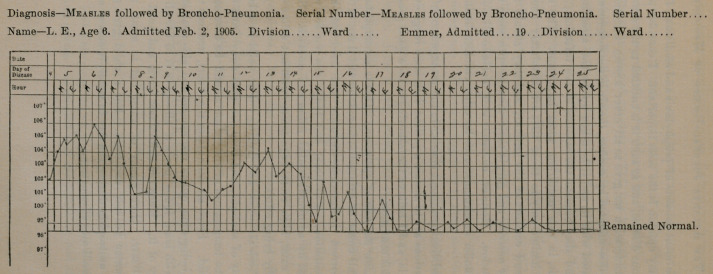# Broncho-Pneumonia in Children

**Published:** 1905-04

**Authors:** Louis C. Rouglin

**Affiliations:** 809-811 English-American Building


ATLANTA
### Journal-Record of Medicine.

**Successor to Atlanta Medical and Surgical Journal, Established 1855, and Southern Medical Record, Established 1870.**

OWNED BY THE ATLANTA MEDICAL JOURNAL CO.

**Published Monthly.**

VOL. VII. APRIL, 1905. No. 1.

BERNARD WOLFF, M.D., EDITOR, Nos. 319-20 Prudential.

M. B. HUTCHINS, M.D., BUSINESS MANAGER, 1007-1008 Century Bldg.

J. N. LECONTE, M.D., ASSOCIATE EDITOR.

### *ORIGINAL COMMUNICATIONS.*

### BRONCHO-PNEUMONIA IN CHILDREN.

BY LOUIS C. ROUGLIN, M.D.

Broncho-pneumonia is an acute infectious disease characterized by circumscribed exudative inflammation of the lung, and a general toxemia manifesting itself by fever, restlessness, delirium, disturbed circulation and respiration.

It is essentially the pneumonia of infancy. The anatomical peculiarities of infancy, such as an abundance of loose connective tissue, abundant lymph-spaces, transitional (young embryonal) cells, abundant blood-vessels and unstable nerve-centers all tend to make any pulmonary inflammation in a child under three years to most commonly be broncho-pneumonia.

It occurs both as a primary condition and secondary to other infectious diseases. When occurring as a primary condition it is characterized by the regularity of infection, and when secondary to other diseases it is characterized by the irregularity of infection.

Of the predisposing causes, the following may be considered in order of their importance: Other diseases, malnutrition, unhygienic surroundings, sex and seasons.

The active agents are pneumococcus in the primary form; pneumoccoccus and streptococcus in the secondary variety. The primary form is characterized by regularity, has an abrupt beginning, a short and sharp duration, and the fever ends by crisis.

The secondary variety has an insidious beginning; temperature is remittent and intermittent and the fever ends by lysis.

Diagnosis—BRONCHO-PNEUMONIA. Serial Number....

Name—A. G., Age 19 months. Admitted Nov. 27, 19..

Division.........., Ward......

Case went to hospital—temperature went down by crisis on 17th day.

Without going into the pathology of the lesions, it is important to know that in the primary form the lesions are most usually lobar, while in the secondary form they are scattered.

It has no typical course of symptoms. Early may be mentioned the abrupt onset, which is the toxemia; children suddenly stop playing; nurslings cry and nurse with difficulty; the child feels chilly; coughs; tongue is usually dry and coated; child is pale, nervous and shivering; the child looks sick. Dr. Northrup, in describing his condition to his class, says, "Child is dopy," and I

know of no better word to describe this condition. This may occur in from two hours to half a day; fever may be present from 102° to 106°; we have distributed ratio of pulse and respiration, which will usually approximate 1:3, may even approximate 1:2.

Later we get in addition physical signs; diminished respiratory murmur, dullness on percussion, broncho-vesicular respiration, harsh breathing and bronchial voice. Lobar dullness wedges off with the lung. Along with these we also get abdominal breathing, recession of lower part of thorax, hernes labialis, respiratory grunt, flush on one or both cheeks. The cough is most constant and often distressing. Pain in the chest is not common, and when present is seldom annoying. Cyanosis is common and may be severe in character. Nervous symptoms are variable; gastro-enteric symptoms are common and are of importance. The temperature is not characteristic, as may be seen from the charts recently observed in my own cases.

The diagnosis should not be difficult. The following combination of symptoms are characteristic and are of importance in order named:

*Early*

Abrupt onset

Fever

Dopy

Ratio of respiration to pulse 1:3

} = Broncho-Pneumonia.

Râles

*Later we get*
Diminished respiratory murmur.
Bronchial voice and breathing.
Broncho-vesicular breathing (harsh puerille).
Dullness.
Recession of lower part of chest.
Abdominal breathing.
Flush on one or both cheeks.
Respiratory grunt.

The ratio of pulse and respiration is of diagnostic importance; it should be counted more than one time, and should by preference be observed when child is quiet and asleep.

As to prognosis, authorities agree that under good hygienic surroundings and proper management, the prognosis is favorable in 95 per cent of all cases.

Diagnosis—BRONCHO-PNEUMONIA. Serial Number—BRONCHO-PNEUMONIA. Serial Number......................

Name—A. L., Age 4 years. Admitted Nov. 15, 19. .Division. . . .Ward. . . . Age 4 years. Admitted Nov. 15, 19. .Division. . . .Ward. . .

Temperature Remained Normal.

Diagnosis—MEASLES followed by Broncho-Pneumonia. Serial Number—MEASLES followed by Broncho-Pneumonia. Serial Number....

Name—L. E., Age 6. Admitted Feb. 2, 1905. Division......Ward...... Emmer, Admitted....19...Division......Ward......

The treatment consists of prophylaxis and medicinal, the first of which in my opinion is the most important. I consider pneumonia an infectious disease, and as such it is entitled to the same prophylactic consideration we give to all other known infections. The disease is disseminated by contact, the air in the sick-room becomes contaminated with it, and it is taken up by the attendants of the patient and they become the carriers of the infection; therefore, *isolation* is, in this condition, most important. The attendants should be limited only to those that are absolutely necessary for the ease and comfort of the patient, and they should frequently wash their nostrils and gargle their mouths with some mild antiseptic solution. Restrict, or better still, exclude all outside visitors, and under no circumstances let other children be in the same room.

The medicinal treatment consists in the management of the case; the indications are to sustain a patient with lame lungs overcoming by toxemia, and I believe the following to be a good general guide to meet this condition:

1st. *Clear the field of operation.* This is accomplished by the use of small doses of calomel followed by full doses of castor oil.

2d. Correct indigestion—regulate the food to avoid gas and fermentation. The heart and lungs can not act well when compressed by an overdistended colon and stomach. Should the condition exist, high rectal regulations with hot saline solutions, will usually bring relief.

3d. Fresh air—cool and flowing. Put child in a large room with plenty of sunlight, and keep the eyes away from the bright light. The temperature of the room should be inversely to that of the child. Fresh air stimulates the heart, reddens the blood, improves indigestion, quiets restlessness and aids against toxemia. A child with lame lungs needs it all—give it to her all the time, and plenty of it. Keep the child's feet warm and head cold.

4th. *Water.* Give plenty of water but in small quantities. Let the water be cool and fresh. For a child give about five grains of urotrophin during the day in water; it acts as an antiseptic, and facilitates elimination.

5th. Absolute rest and quiet is essential. Do not bring outside influences to a patient who is nervous, it makes him more so; sleep comforts the patient, it should not be disturbed.

6th. Promote general comfort. This can be accomplished by frequent change to dry, warm garments to avoid maceration of skin, allowing long restful sleep, by not too frequent feedings and dosing, by sponging the face and wetting the lips, and by the doctor and attendants being congenial and tactful.

7th. Heart stimulants. When the gas collecting in the stomach will be properly cared for, heart stimulants will, as a rule, not be indicated; when indicated, whisky, strychnine, one or both judiciously given will meet the indication.

8th. Make use of sponging or cold applications of water to reduce temperature. Use no antipyretics to allay nervousness. A grain of phenacetin may occasionally be given; coal-tar preparations should not be used for the fever. When pain is present in the chest, an application of antiphlogistin applied warm and thick will relieve it. I use it only when pain is complained of. A hot foot-bath will usually insure a good night's rest. Use no opium for the cough when distressing. Creosote by inhalation will suffice and is preferable to internal medication.

9th. *Avoid* overcrowding of room, constriction of chest, swathing poultices, maceration of child's skin, disturbing sleep, over-feeding and over-drugging, and above all avoid opium and coal-tar products. Treat the case the same as you would a case of toxemia occurring elsewhere in the body and the results will in all possible cases be favorable.

*809-811 English-American Building.*

## Figures and Tables

**Figure f1:**
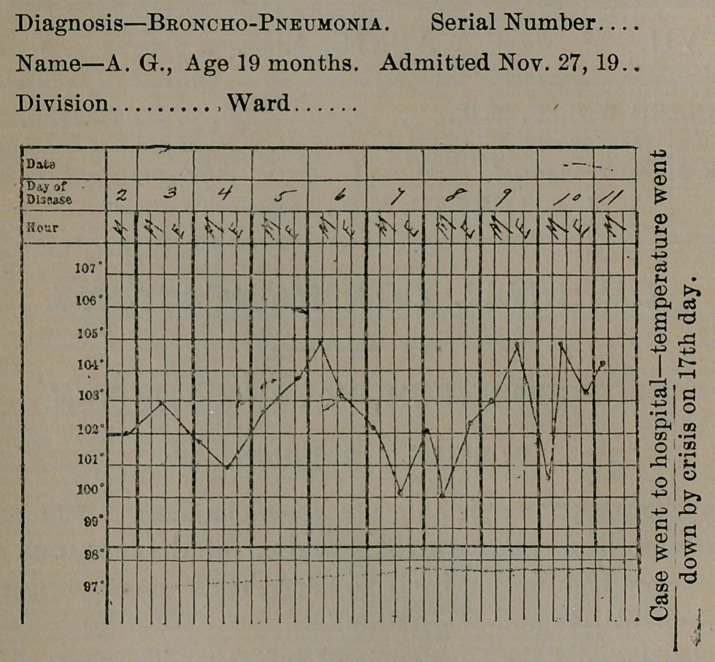


**Figure f2:**
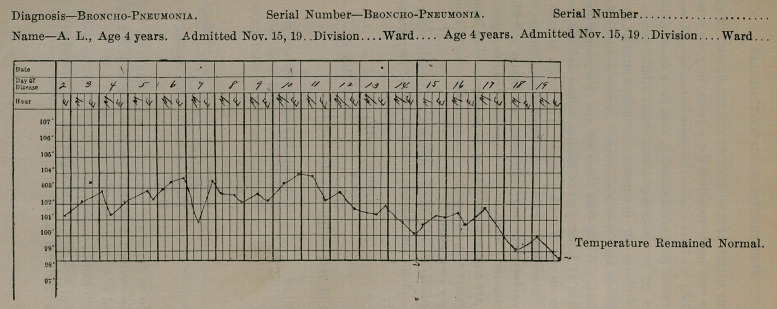


**Figure f3:**